# 1377. Rabies and Tetanus Post-Exposure Prophylaxis Initiation in the US and Overseas: Consistent with ACIP Recommendations?

**DOI:** 10.1093/ofid/ofad500.1214

**Published:** 2023-11-27

**Authors:** Siri L Chirumamilla, Jennifer Breiman, Jessica K Fairley, Jesse J Wagonner, Matthew H Collins, Henry M Wu

**Affiliations:** Emory University, Atlanta, Georgia; Emory Healthcare, Atlanta, Georgia; Emory University, Division of Infectious Diseases, Atlanta, Georgia; Emory University, Atlanta, Georgia; Emory University, Atlanta, Georgia; Emory University, Atlanta, Georgia

## Abstract

**Background:**

Appropriate management of animal bites is critical to prevent rabies encephalitis and tetanus. In the US, rabies post-exposure prophylaxis (PEP) is typically started in emergency departments (EDs). For travelers seeking care overseas, World Health Organization (WHO) PEP guidance differs from those of the Advisory Council on Immunization Practices (ACIP). WHO does not recommend rabies immune globulin (RIG) for minor scratches; furthermore, RIG availability can vary worldwide. Outpatient follow-up of patients not given RIG is challenging because RIG is often not given in clinics. We assessed whether patients referred to a travel medicine clinic for rabies PEP vaccination were initially managed in accordance with ACIP guidance.

**Methods:**

Medical records of patients presenting for rabies PEP at the Emory TravelWell Center from 2017-21 were reviewed. Initial management after exposure at the patient’s first healthcare interaction was assessed for consistency with ACIP recommendations for rabies and tetanus PEP.

**Results:**

Fifty-six patients were seen for rabies PEP from 2017-21. The median age was 38 years (range 5 to 67). Most patients were exposed in the US (71%). Animal bites accounted for most exposures (57%), followed by suspected contact without visible bite or scratch (38%), and scratches (5%). Frequently involved animals were bats (41%), dogs (30%), cats (14%), and monkeys (7%). A tetanus booster was indicated but not given in 59% of patients. Most patients (82%) received ACIP-consistent rabies PEP. Among the 10 patients who did not, seven (70%) had initiated PEP overseas without RIG. Three patients who initiated PEP in the US received PEP that was inconsistent with ACIP guidance; two did not receive RIG and one received gluteal vaccination. None developed symptomatic rabies or tetanus infection.

**Conclusion:**

Patients seen at a travel clinic for rabies PEP often start it elsewhere, and in this series 18% of patients had PEP initiation inconsistent with ACIP guidance. Returned travelers who initiated PEP in accordance with WHO guidance account for most situations where RIG was not given. Errors in rabies and tetanus PEP also occurred in US EDs. US providers administering rabies and tetanus PEP should ensure practices adhere to ACIP guidance.

**Disclosures:**

**All Authors**: No reported disclosures

